# Diagnostic and prognostic predictive values of triggering receptor expressed on myeloid cell-1 expression in neonatal sepsis: A meta-analysis and systematic review

**DOI:** 10.3389/fped.2022.929665

**Published:** 2022-07-22

**Authors:** Chenyang Chang, Qiannan Gao, Guoping Deng, Kaiyuan Luo, Huifang Zhu

**Affiliations:** ^1^The First Clinical Medical College of Gannan Medical University, Ganzhou, China; ^2^Pediatric Internal Medicine, Children's Medical Center, First Affiliated Hospital of Gannan Medical University, Ganzhou, China; ^3^Neonatal/Pediatric Intensive Care Unit, Children's Medical Center, First Affiliated Hospital of Gannan Medical University, Ganzhou, China; ^4^Institute of Children's Medical, First Affiliated Hospital of Gannan Medical University, Ganzhou, China; ^5^Ganzhou Key Laboratory of Immunotherapeutic Drugs Developing for Childhood Leukemia, Ganzhou, China; ^6^Basic Medical College of Gannan Medical University, Ganzhou, China; ^7^Department of Pathogenic Biology, School of Basic Medical Sciences, Gannan Medical University, Ganzhou, China

**Keywords:** TREM-1, STREM-1, neonatal sepsis, diagnosis, prognosis

## Abstract

**Objective:**

The purpose of this systematic review was to explore the value of the expression level of the triggering receptor expressed on myeloid cell-1 (TREM-1) in the diagnosis and prognosis of neonatal sepsis.

**Methods:**

A comprehensive search was performed to identify the diagnostic and prognostic predictive values of the TREM-1 expression level in neonatal sepsis. Based on the retrieval strategy, Cochrane Library, Embase, Ovid, ProQuest, PubMed, Scopus, and Web of Science databases were searched from inception to February 2022. Studies were included if they assessed the accuracy of TREM-1 expression in the diagnosis of neonatal sepsis and distinguished survival and death in neonatal sepsis. Two authors independently evaluated the study and extracted the data, including the first author of the literature, country, total study population, basic population characteristics of the study group and the control group, study design (observational studies), type of sample, sepsis onset, type of biomarker, assay method, cut-off, sensitivity, specificity, true positives (TP), false positives (FP), false negatives (FN), and true negatives (TN). A third party will be consulted if disputed. The accuracy of TREM-1 expression in the diagnosis and prognostic prediction of neonatal sepsis was evaluated by a bivariate mixed-effects model. The source of heterogeneity was explored through meta-regression analysis.

**Results:**

Thirteen articles that met the research criteria were included in qualitative analysis, and 11 of them were included in quantitative analysis. The pooled sensitivity, specificity, positive likelihood ratio (PLR), negative likelihood ratio (NLR), diagnostic odds ratio (DOR), and the area under the summary receiver operator characteristic (SROC) curve of soluble TREM-1 (sTREM-1) were 0.94 (95% CI: 0.82, 0.98), 0.87 (95% CI: 0.70, 0.95), 7.36 (95% CI: 2.75, 19.74), 0.07 (95% CI: 0.02, 0.24), 111.71 (95% CI: 13.24, 942.92), and 0.96 (95% CI: 0.94, 0.98), respectively. Meta-regression and subgroup analysis were used to investigate the heterogeneity, owing to non-threshold effects caused by types of test sample and research design. sTREM-1 as a biomarker for distinguishing survival and death in neonates with sepsis had pooled sensitivity, specificity, area under the SROC curve, PLR, NLR, and DOR of 0.95 (95% CI: 0.83, 0.99), 0.98 (95% CI: 0.68, 1.00), 0.99 (95% CI: 0.97, 0.99), 39.28 (95% CI: 2.13, 723.99), 0.05 (95% CI: 0.01, 0.19), and 789.61 (95% CI: 17.53, 35,560.72), respectively.

**Conclusion:**

The study showed that TREM-1 was a potential biomarker for the diagnosis and prognosis of neonatal sepsis. The biggest advantage of this study is that it is the first to comprehensively explore the role of TREM-1 expression in the diagnosis and prognosis of neonatal sepsis. However, there are some limitations in this study, such as the reduced number of clinical studies on TREM-1 expression as a biomarker of neonatal sepsis, regional bias, and differences in detection methods. Hence, more large-scale and high-quality studies are needed to improve diagnostic accuracy.

**Systematic review registration:**

https://www.crd.york.ac.uk/PROSPERO/, identifier: CRD42022338041.

## Introduction

Neonatal sepsis is considered to be a systemic inflammatory response syndrome (SIRS) induced by bacteria, viruses, or fungi (yeast) infections (1). It can be divided into early-onset sepsis (EOS, ≤ 72 h of age) and late-onset sepsis (LOS, >72 h of age) depending on the onset time (2, 3). Neonatal sepsis is a major disease threatening the life of newborns, which is also one of the major challenges facing global public health. The occurrence of neonatal sepsis was found in 2,202 newborns per 100,000 live births, with a mortality rate of 11–19% (4). In 2015, 336,300 newborns died of neonatal sepsis, accounting for 12.8 % of neonatal deaths (5). Therefore, the timely diagnosis and effective evaluation of the prognosis of neonatal sepsis is particularly important, which may greatly and effectively reduce its morbidity and mortality.

To date, blood culture is still considered as the “-Gold Standard-” for the diagnosis of neonatal sepsis, but the results are often delayed for 24–48 h and vulnerability to multiple factors, which may greatly limit its application to the early diagnosis and treatment of neonatal sepsis (6). Moreover, laboratory indicators available including white blood cell count, immature neutrophil to total neutrophil ratio, C-reactive protein, etc., are not fully applicable to the diagnosis of neonatal sepsis (1, 2, 7). Besides, the clinical symptoms and signs of neonatal sepsis are often non-specific (e.g., unstable temperature, hypotension, metabolic acidosis, tachycardia or bradycardia, apnea, etc.), making it easy to miss the optimal treatment opportunity and usually leading to adverse outcomes (1). Therefore, it is urgent to find valuable biomarkers for the early diagnosis of neonatal sepsis.

The triggering receptor expressed on myeloid cell-1 (TREM-1) is a novel transmembrane protein discovered by Swiss researchers in 2000, which is a new receptor of the immunoglobulin superfamily (8). It is expressed both on the surface of immune cells (e.g., neutrophils, monocytes, macrophages, T lymphocytes, B lymphocytes, natural killer cells, monocyte-derived dendritic cells, etc.) (8–10) and on non-immune cells (e.g., gastric epithelial cells, liver endothelial cells, etc.) (11, 12). TREM-1 exists in two forms: membrane-bound TREM-1 (mTREM-1) and soluble TREM-1 (sTREM-1). When binding to its ligand, mTREM-1 can promote cell activation through an associated signal transduction molecule DNAX activation protein of 12 kDa (DAP12), leading to tyrosine phosphorylation in the immunoreceptor tyrosine-based activation motif (ITAM) chain and providing a binding site, spleen tyrosine kinase (SYK) and zeta-chain-associated protein kinase 70 (ZAP70) (13–18). After that, SYK regulates the activation of downstream signaling pathways and ultimately induces the activation of transcription factors, which in turn regulates the expression of inflammatory responses (18–20). TREM-1 plays multiple roles in regulating the host's antimicrobial immune responses, on the one hand, it can cooperate with toll-like receptor (TLR) to promote the release of proinflammatory cytokines/chemokines upon the infection of *Mycobacterium tuberculosis*, which contributes to the host defense against microbial challenges during both the early-induced and adaptive phases (21), and on the other hand, the release of sTREM-1 was also reported to be an amplifier of the SIRS associated with sepsis (22). Currently, TREM-1 is considered a potential therapeutic target in inflammatory diseases (15, 17). It was demonstrated that a fusion protein named mTREM-1/IgG1 containing murine TREM-1 and human IgG1 Fc played a protective role in mice with septic shock induced by lipopolysaccharide (LPS), lethal *E. coli*, or cecum ligation and puncture (CLP) (23). The production of proinflammatory cytokines and infiltration of neutrophils or monocytes/macrophages into the peritoneum were significantly reduced upon the pretreatment of TREM-1/IgG1 in LPS injection mice compared with controls (23). It is suggested that TREM-1 is closely related to the occurrence and development of inflammatory responses.

Two meta-analyses revealed that sTREM-1 had a moderate ability (area under curve (AUC): 0.89 and 0.88, respectively) to diagnose sepsis in adults (24, 25). sTREM-1 had a certain diagnostic value not only for adult sepsis but also for neonatal sepsis. A previous meta-analysis suggested that sTREM-1 might be a useful biomarker for predicting neonatal sepsis (26). However, this study might have overestimated the actual predictive ability of sTREM-1 in diagnosing neonatal sepsis, owing to the lack of analysis of the heterogeneity (26). At the same time, not all of the participants in one of the studies included were 28 days or younger (27). In recent years, with the novel discovery of the role of TREM-1 expression in neonatal sepsis, an update of its diagnostic values is extremely necessary to meet the demands of clinical work. In this study, we had enlarged the sample size and explored the source of potential heterogeneity. In addition, the mRNA transcription and protein expression level of TREM-1 on the cell surface were qualitatively analyzed in the diagnostic value of neonatal sepsis. Moreover, meta-analyses showed that sTREM-1 also had a moderate predictive capability (AUC: 0.82 and 0.78, respectively) in assessing adult sepsis mortality (25, 28); however, there was no involved data to evaluate the prognostic value of TREM-1 expression in neonatal sepsis in those studies. We hope to investigate the application of TREM-1 expression in clinical practice. Therefore, it was necessary to quantitatively and qualitatively analyze the value of TREM-1 expression in the diagnosis and prognosis of neonatal sepsis.

## Methods

The present study was conducted based on the Preferred Reporting Items for Systematic Reviews and Meta-Analyses (PRISMA) guidelines (29). The study has also been registered in PROSPERO (CRD42022338041).

### Search strategy

Databases including the Cochrane Library, Embase, Ovid, ProQuest, PubMed, Scopus, and Web of science were searched from inception to February 2022 for the role of TREM-1 expression in the diagnosis and prognosis of neonatal sepsis. Our search strategy used the following keywords: “neonatal sepsis,” “newborn,” “septic,” “septicemia,” “newborn sepsis,” “triggering receptor expressed on myeloid cells-1,” “TREM-1,” “soluble triggering expressed receptor on myeloid cells-1,” “sTREM-1,” “TREM-1 protein, human.” Search strategies for all databases are shown in Supplementary Material. Meanwhile, we obtained relevant literature through other means, such as a list of references to obtained articles.

### Study selection

The inclusion criteria were as follows: (1) the purpose of the study was to evaluate the value of TREM-1 expression (including sTREM-1, mTREM-1, and TREM-1 mRNA) as a biomarker in the diagnosis and/or distinguishing survival and death of neonatal sepsis; (2) a 2 × 2 contingency table containing TP, FP, FN, and TN could be obtained; (3) participants: newborns. Subjects were culture-positive and/or clinically diagnosed with sepsis; (4) study types: relevant clinical observational studies (both prospective and retrospective).

Studies were excluded based on the following criteria: (1) review, editorial, commentary, research protocol, animal experiments, case reports, meta-analysis, and systematic review; (2) articles not published in English; (3) patients older than 28 days (e.g., children, adolescents, and adults); (4) repeated articles; (5) incomplete reporting of original data or the inability to obtain full-text literature.

Two researchers (QG and GD) independently screened the literature according to inclusion criteria and exclusion criteria. First, a preliminary screening was conducted by reading the title and abstract. After excluding irrelevant literature, the remaining were further screened by reading the full text to determine the final included literature. In case of disagreement between two researchers (QG and GD), a third party (HZ) could be invited for a consultation to ensure the reliability of included literature and reduce publication bias and heterogeneity.

### Data extraction and quality assessment

Two researchers (QG and GD) extracted literature data, including the first author of the literature, country, total study population, basic population characteristics (gestational age, birth weight, gender) of the study group and the control group, study design, type of sample, sepsis onset, type of biomarker, assay method, cut-off, sensitivity, specificity, TP, FP, FN, and TN. For literature with incomplete data, it was tried to be obtained directly from the original author by email. If no response was received after sending the reminder, the literature was excluded.

We evaluated the risk of bias in research and diagnostic criterion suitability by using the Quality Assessment of Diagnostic Accuracy Studies (QUADAS-2 tool) (30). QUADAS-2 consisted of four parts: patient selection, index test, reference standard, and flow and timing (30). These four items were used to assess the risk of bias in the included literature, and the first 3 items could also be used to evaluate clinical applicability. For specific evaluation criteria, refer to the references (30). Quality assessment was carried out by two researchers (QG and GD) and negotiated by a third party (HZ) in case of disagreement.

### Statistical analysis

The quality evaluation results of the included literature were drawn by RevMan 5.3 software. MIDAS module of STATA16.0 was used for statistical analysis, and *P* < 0.05 was considered statistically significant. Sensitivity, specificity, DOR, PLR, NLR, and SROC were analyzed and summarized using the bivariate mixed-effects model (31). The overall diagnostic performance of sTREM-1 in the diagnosis and prognosis of neonatal sepsis was measured by the SROC curve (32). Heterogeneity between studies was assessed by *Q*-test and *I*^2^ index (33, 34). When *P*-value of *Q*-test < 0.05 and *I*^2^ index ≥50%, moderate heterogeneity existed, and the source of heterogeneity needed to be discussed. In addition to the proportion of heterogeneity that might be due to threshold effects, univariate meta-regression analysis and subgroup analysis (including type of sample, study design, and sample size) were used to explore the sources of potential heterogeneity. Cook's Distance was used for sensitivity analysis to assess the stability of the study results. Deek's funnel plot asymmetry test was drawn to evaluate the publication bias of the included literature (35). If the result of Deek's symmetry test was *P* < 0.05, publication bias was suggested.

## Results

**Figure 1:**
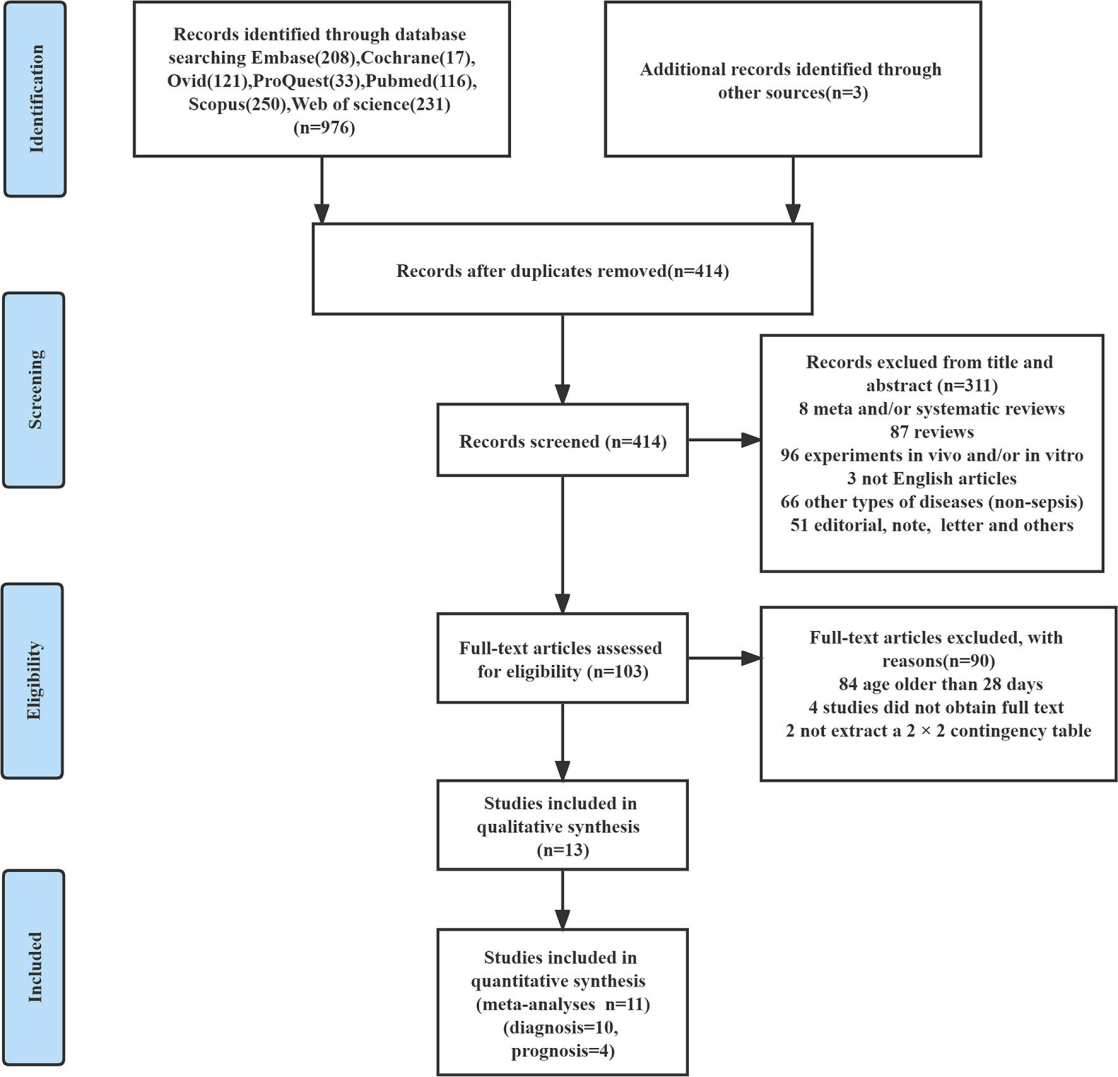
The detailed flow chart for study selection.

According to the literature retrieval strategy set in this study, 976 related literature were retrieved, and three related literature were obtained through other means, totaling 979 literature. By reading the titles and abstracts of included literature and eliminating duplicate literature, we finally re-screened the remaining 103 literature and read them in full. Among them, 84 studies included patients older than 28 days, four studies could not obtain full-texts, and two studies could not extract a 2 × 2 contingency table. Finally, 13 studies (36–48) that met our research criteria were included in qualitative analysis and 11 studies (36, 38–47) were included in quantitative analysis. The literature screening process is shown in Figure 1.

### Characteristics of included studies

The 13 included studies were published between 2010 and 2021, and all were published in English (36–48). Among them, 12 studies discussed the characteristics of articles on the diagnostic value of TREM-1 expression for neonatal sepsis (Table 1) (36–39, 41–48). Table 2 showed the data related to the diagnostic accuracy of TREM-1 expression in neonatal sepsis. The expression of TREM-1 was sTREM-1 in 10 studies (36, 38, 39, 41–47), one about TREM-1 mRNA (48), and another one about mTREM-1 (37). Six studies were conducted in Egypt (39, 41, 43, 44, 46, 48), three in Turkey (42, 45, 47), and the remaining three studies were conducted in European countries (Italy, Switzerland, and Greece) (36–38). The study group included newborns with EOS in one study (38), newborns with LOS in six studies (36, 37, 42, 43, 45, 47), EOS+LOS in four studies (39, 44, 46, 48), and one study was not mentioned (41). Ten studies measured TREM-1 expression levels by enzyme-linked immunosorbent assay (ELISA) (36, 38, 39, 41–47), one by flow cytometry (37), and another one by reverse transcription-polymerase chain reaction (RT-PCR) (48). Considering the different detection methods, 10 studies were included in the meta-analysis, with a total of 743 newborns (36, 38, 39, 41–47).

**Table 1:** Characteristics of the included studies that investigated the diagnostic value of TREM-1 expression in neonatal sepsis.

**References**	**Country**	**No. of cases**	**Patients/control (** ***n*** **)**	**GA (weeks)**	**BW (g)**	**Sex male %**	**Study design**	**Type of sample**	**Onset**
Sarafidis et al. (36)	Greece	52	31 infected neonates/ 21 non-infected newborns	35 (24–40)/30 (24–39)	1,990 (890–3,600)/1,190 (720–4,785)	61.29/57.14	Prospective	Serum	LOS
Schlapbach et al. (38)	Switzerland	137	33 infected neonates/ 104 non-infected newborns	39.9 (34.0–41.6)/38.9 (34.0–42.0)	3,335 (1,950–4,400)/3,060(1,630–4,750)	54.55/65.38	Prospective	Serum	EOS
Mazzucchelli et al. (37)	Italy	32	16 septic group/16 control group	27.5 ± 2.7/26.9 ± 1.0	1,055 ± 492/1,155 ± 211	56.25/43.75	Case-control	Whole blood	LOS
Adly et al. (39)	Egypt	152	112 neonatal sepsis/ 40 healthy controls	N/A	N/A	52.68/55	Prospective	Serum	EOS+LOS
Saldir et al. (42)	Turkey	50	30 septic group/20 non-septic group	37.9 ± 1.7/38.3 ± 1.6	3,201 ± 313/3,328 ± 383	40/55	Prospective	Serum	LOS
El-Gendy et al. (41)	Egypt	60	40 neonatal sepsis/ 20 healthy controls	35.8 ± 2.94/36.5 ± 2.31	2,580 ± 640/2,910 ± 610	52.5/50	Case-control	Serum	N/A
El-Khier et al. (43)	Egypt	59	30 sepsis group/ 29 control group	31.7 ± 3.1/32 ± 3.1	1,520 (1,117.5–1,925)/1,470 (1,090–2,450)	43.33/55.17	Prospective	Serum	LOS
Zidan et al. (44)	Egypt	45	35 septic neonates/10 healthy newborns	N/A	N/A	N/A	Prospective	Serum	EOS+LOS
Alkan Ozdemir et al. (45)	Turkey	62	31 septic group/31 control group	28.6 ± 3.2/29.7 ± 3.0	1,114 ± 439/1,226 ± 382	58.06/54.84	Prospective	Urine	LOS
El-Madbouly et al. (46)	Egypt	60	30 septic group/ 30 control group	37.9 ± 1.7/39 ± 1.4	2,900 ± 700/3,000 ± 300	96.7/86.7	Prospective	Serum	EOS+LOS
Ozdemir et al. (47)	Turkey	66	31 septic group/ 35 control group	31.9 ± 5.0/34.1 ± 4.6	1,771 ± 1,069/2,190 ± 1,015	70.97/62.86	Prospective	Urine	LOS
Ghonaim et al. (48)	Egypt	100	75 neonatal sepsis/25 healthy newborns	N/A	N/A	54.67/56	Case-control	Whole blood	EOS+LOS

*GA, gestational age; BW, birth weight; EOS, early onset sepsis; LOS, late onset sepsis; N/A, not applicable; No, number*.

**Table 2:** The data related to the diagnostic accuracy of TREM-1 expression in neonatal sepsis.

**References**	**Biomarkers**	**Assay method**	**Cut-off**	**Sensitivity/ Specificity (%)**	**TP**	**FP**	**FN**	**TN**	**AUC**
Sarafidis et al. (36)	sTREM-1	ELISA	143.35 Pg /ml	70/71	22	6	9	15	0.733
Schlapbach et al. (38)	sTREM-1	ELISA	1,250 Pg /ml	75/52	25	50	8	54	0.62
Mazzucchelli et al. (37)	mTREM-1	Routine flow cytometry	62.12%	56.2/93.5	9	1	7	15	0.8
Adly et al. (39)	sTREM-1	ELISA	310 Pg /ml	100/100	112	0	0	40	1
Saldir et al. (42)	sTREM-1	ELISA	450 Pg /ml	93.3/90	28	2	2	18	0.97
El-Gendy et al. (41)	sTREM-1	ELISA	1,707.35 Pg /ml	100/100	40	0	0	20	1
El-Khier et al. (43)	sTREM-1	ELISA	77.5 Pg /ml	90/51.7	27	14	3	15	N/A
Zidan et al. (44)	sTREM-1	ELISA	250 Pg /ml	97.1/90	34	1	1	9	0.97
Ozdemir et al. (45)	sTREM-1	ELISA	78.5 Pg /ml	90/78	28	7	3	24	0.87
El-Madbouly et al. (46)	sTREM-1	ELISA	69.8 Pg /ml	96.7/86.7	29	4	1	26	N/A
Alkan Ozdemir et al. (47)	sTREM-1	ELISA	129 Pg /ml	63.64/84.85	20	5	11	30	N/A
Ghonaim et al. (48)	• TREM-1 • mRNA	RT-PCR	0.631	65.33/96	49	1	26	24	0.708

*TREM-1, triggering receptor expressed on myeloid cell-1; sTREM-1, soluble triggering expressed receptor on myeloid cells-1; ELISA, enzyme-linked immunosorbent assay; TP, true positives; FP, false positives; FN, false negatives; TN, true negatives; RT-PCR, reverse transcription-polymerase chain reaction*.

Table 3 reported the characteristics of five studies that explored the prognostic value of TREM-1 expression in neonatal sepsis (39–41, 43, 48). Among them, the expression of TREM-1 was sTREM-1 in four studies (39–41, 43) and TREM-1 mRNA was expressed in the other study (48). Four studies (39, 41, 43, 48) were conducted in Egypt and one (40) in Mexico. The study group in two studies was newborns with LOS (40, 43), newborns with EOS+LOS in two studies (39, 48), and the study group was not mentioned in one study (41). TREM-1 expression was measured by ELISA in four studies (39–41, 43) and by RT-PCR in one (48). Due to different detection methods, four studies were included in the meta-analysis, with a total of 204 newborns (39–41, 43).

**Table 3:** Characteristics of the included studies that explored the prognostic value of TREM-1 expression in neonatal sepsis.

**References**	**Country**	**No**.	**Survivors/non- survivors (** ***n*** **)**	**Study design**	**Type of sample**	**Onset**	**Biomarkers**	**Assay method**	**Cut-off**	**Sensitivity/ specificity (%)**	**TP**	**FP**	**FN**	**TN**	**AUC**
Adly et al. (39)	Egypt	63	47/16	Prospective	Serum	EOS+LOS	sTREM-1	ELISA	1,100 Pg /ml	100/97	47	0	0	16	0.978
El-Gendy et al. (41)	Egypt	40	23/17	Case-control	Serum	N/A	sTREM-1	ELISA	2,902.2 Pg/ml	88.2/78.3	20	4	3	13	0.92
Arízaga-Ballesteros et al. (40)	Mexico	71	62/9	Cross-sectional	Plasma	LOS	sTREM-1	ELISA	300 Pg/ml	97/78	60	2	2	7	0.884
El-Khier et al. (43)	Egypt	30	27/3	Prospective	Serum	LOS	sTREM-1	ELISA	91.5 Pg /ml	96.3/100	26	0	1	3	0.988
Ghonaim et al. (48)	Egypt	75	34/41	Case-control	Whole blood	EOS+LOS	TREM-1 mRNA	RT-PCR	0.369	87.8/97.06	30	1	4	40	0.902

*TREM-1, triggering receptor expressed on myeloid cell-1; sTREM-1, soluble triggering expressed receptor on myeloid cells-1; ELISA, enzyme-linked immunosorbent assay; RT-PCR, reverse transcription-polymerase chain reaction; TP, true positives; FP, false positives; FN, false negatives; TN, true negatives; AUC, area under the curve; N/A, not applicable; No, number*.

### Quality assessment

**Figure 2:**
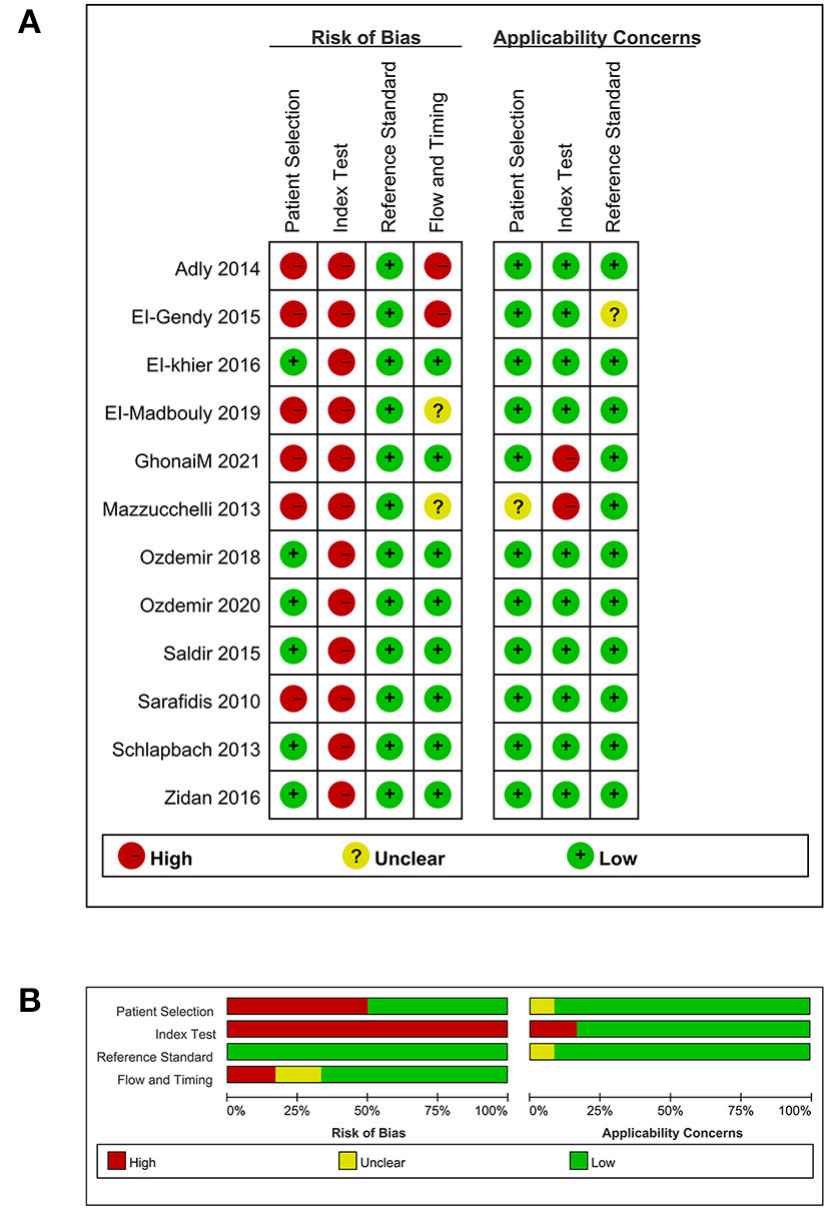
Quality assessment. **(A)** Methodological quality summary. **(B)** Methodological quality graph.

Since one study did not involve the diagnostic value of TREM-1 expression in neonatal sepsis (40), we only evaluated the quality of the remaining 12 studies (36–39, 41–48). The results of the assessment of bias risk and suitability of the remaining 12 studies is shown in Figure 2. The reason why a high risk of bias was detected in the domain of index test was that the threshold value for the detection of neonatal sepsis by TREM-1 expression was not predetermined, but determined by the receiver operating characteristic (ROC) curve. The overall bias risk of the 12 studies was within an acceptable range, with high applicability and in line with QUADAS-2 evaluation criteria.

### Data analysis in diagnostic value

**Figure 3:**
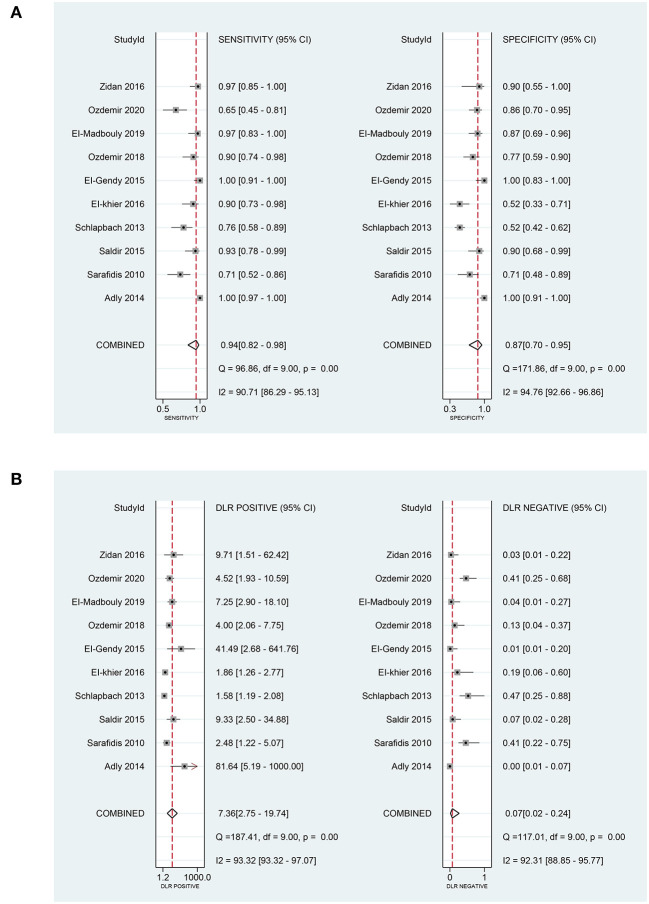
The pooled sensitivity, specificity, PLR, and NLR of sTREM-1 in diagnosing neonatal sepsis. **(A)** The pooled sensitivity and specificity. **(B)** The pooled PLR and NLR. PLR, positive likelihood ratio; NLR, negative likelihood ratio; sTREM-1, soluble triggering receptor expressed on myeloid cell-1.

**Figure 4:**
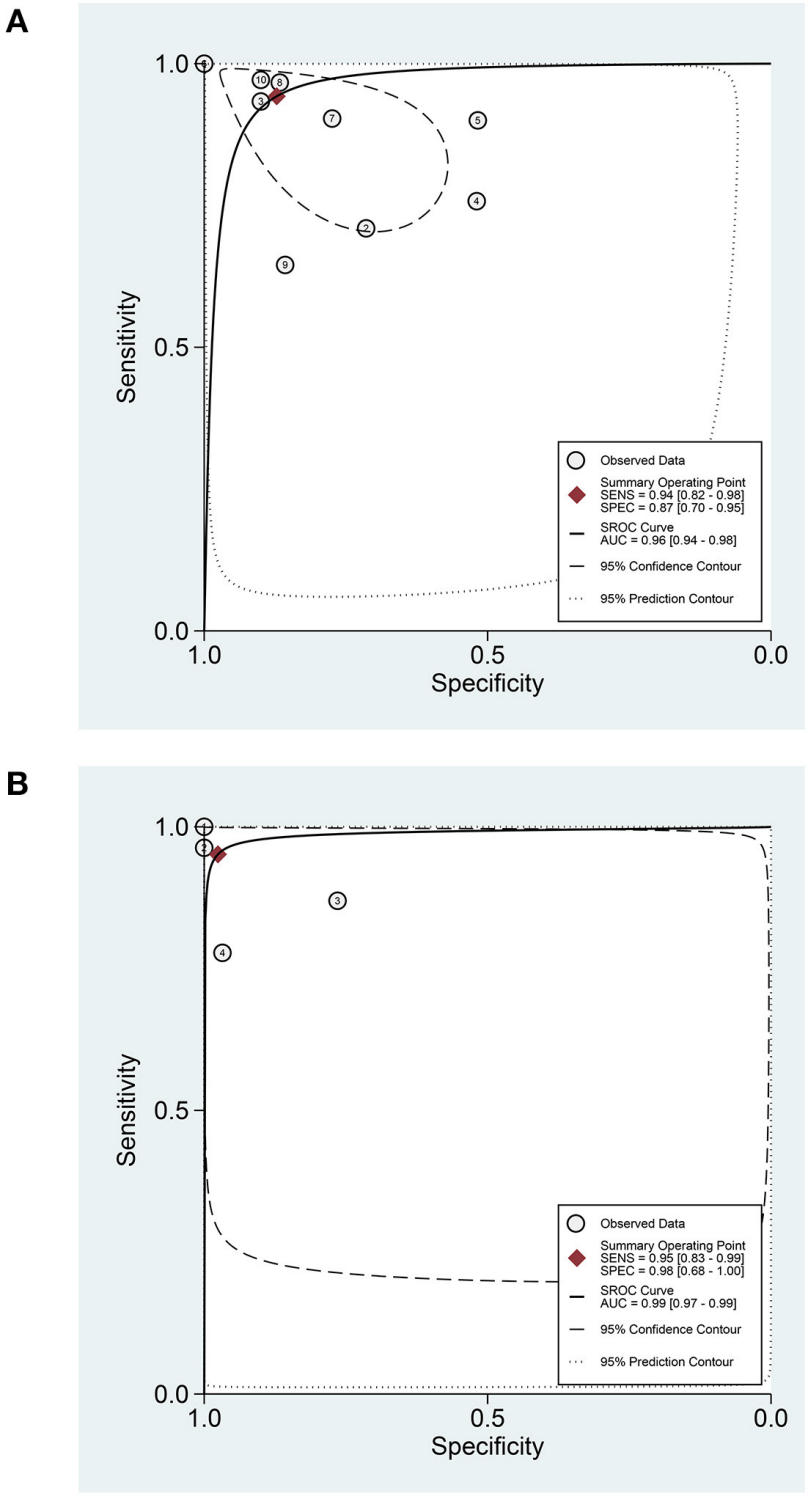
The SROC curve of sTREM-1 in diagnosing neonatal sepsis and distinguishing survival and death in neonatal sepsis. **(A)** In diagnosing neonatal sepsis. **(B)** In distinguishing survival and death in neonatal sepsis. sTREM-1, soluble triggering receptor expressed on myeloid cell-1; SROC, summary receiver operator characteristic.

**Figure 5:**
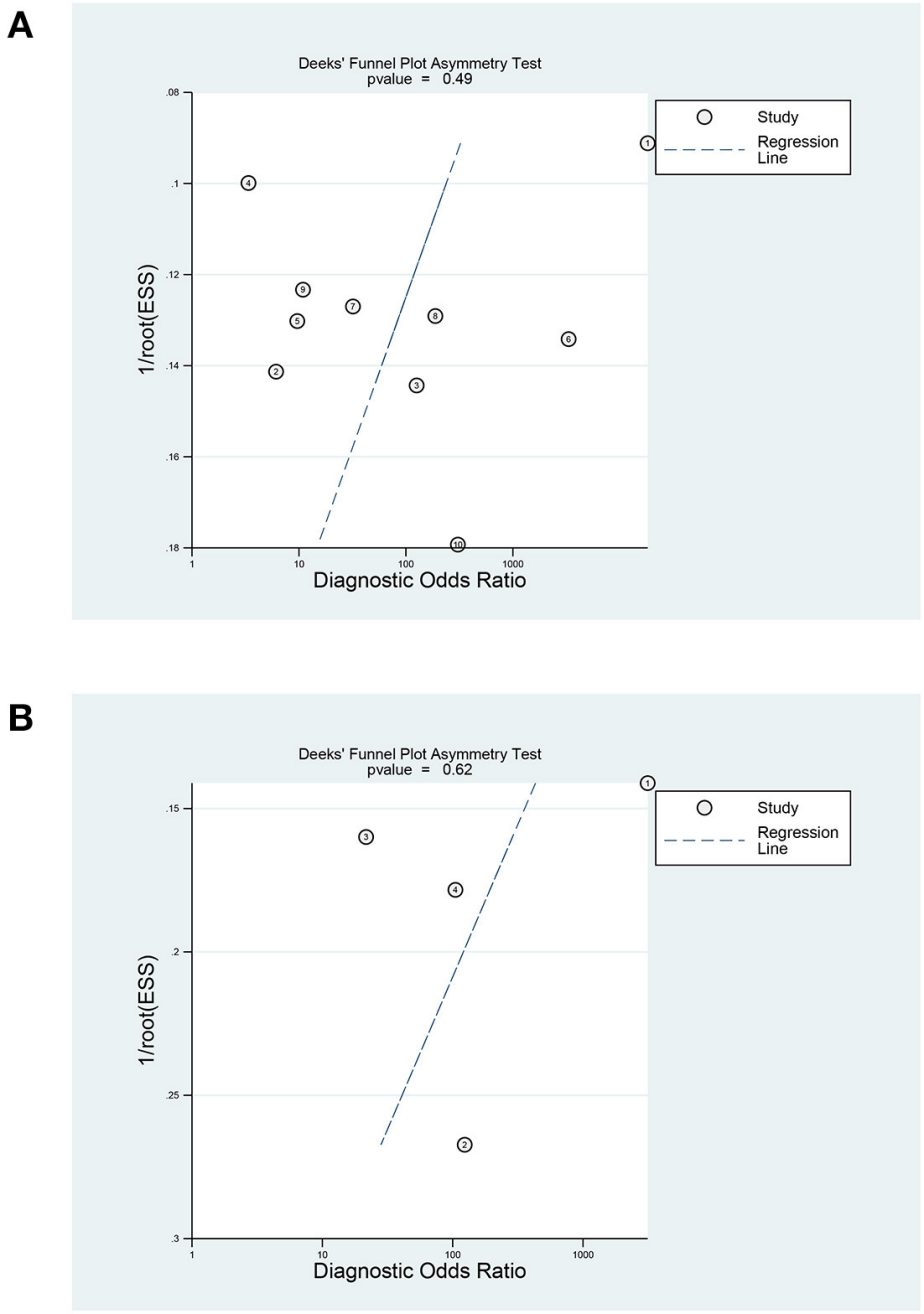
Deeks' funnel plot of sTREM-1 in diagnosing neonatal sepsis and distinguishing survival and death in neonatal sepsis. **(A)** In diagnosing neonatal sepsis. **(B)** In distinguishing survival and death in neonatal sepsis. sTREM-1, soluble triggering receptor expressed on myeloid cell-1.

In terms of the diagnostic value of sTREM-1 in neonatal sepsis, the aggregate sensitivity and specificity were 0.94 (95% CI: 0.82, 0.98) and 0.87 (95% CI: 0.70, 0.95), respectively (Figure 3A). The pooled PLR and NLR were 7.36 (95% CI: 2.75, 19.74) and 0.07 (95% CI: 0.02, 0.24), respectively (Figure 3B). The DOR was 111.71 (95% CI: 13.24, 942.92) and the area under the SROC curve was 0.96 (95% CI: 0.94, 0.98; Figure 4A). Deek's funnel plot asymmetry test was used to evaluate publication bias, and the *P* = 0.49, indicating that the funnel plot had no obvious asymmetry or publication bias, which further indicated that the results of this meta-analysis were reliable (Figure 5A).

**Figure 6:**
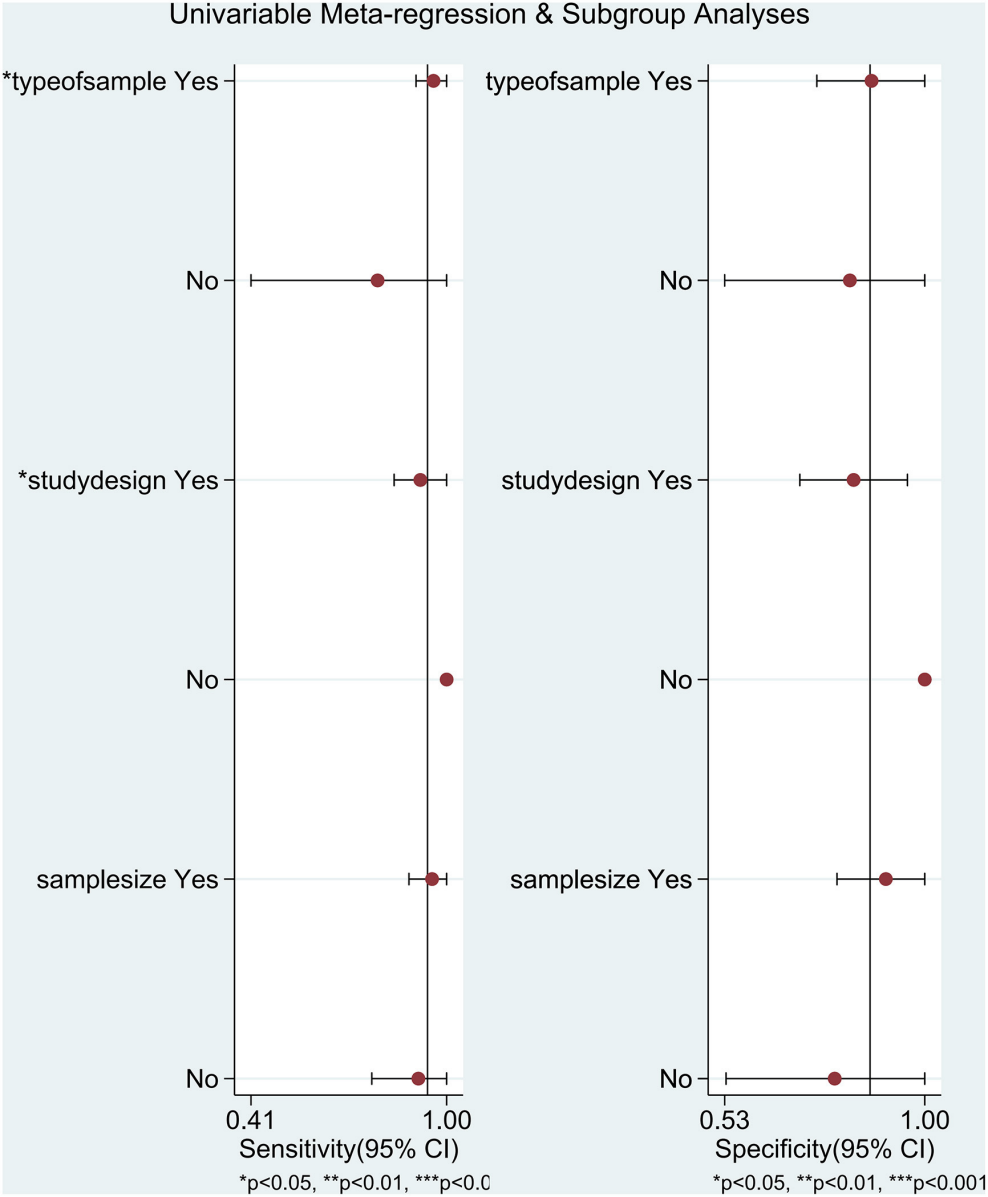
Univariate meta-regression analysis and subgroup analysis in diagnosing neonatal sepsis. **P < 0.01, ***P < 0.001.

There was significant heterogeneity among studies (overall *I*^2^ for bivariate mixed-effects model 63%, 95% CI: 17, 100). The *I*^2^-test results for the pooled sensitivity and specificity were 90.71% (*P* < 0.05) and 94.76% (*P* < 0.05). The proportion of heterogeneity that could be caused by the threshold effect was 0.76. Due to testing using STATA's MIDAS module, we did not record evidence of threshold effects. To explore the source of potential heterogeneity, we used univariate meta-regression analysis and subgroup analysis. The covariables of meta-regression included type of sample (blood sample or not), study design (prospective study or not), and sample size (≥60 newborns or not). We found that type of sample and study design was possibly correlated with the heterogeneity of sensitivity (Figure 6). The results of the subgroup analysis are shown in Supplementary Table 1.

**Figure 7:**
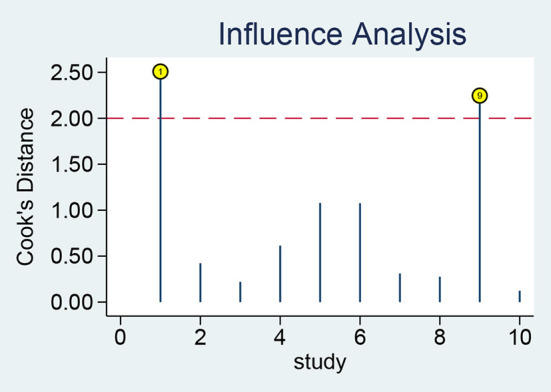
Sensitivity analysis of sTREM-1 in diagnosing neonatal sepsis. sTREM-1, soluble triggering receptor expressed on myeloid cell-1.

The results of sensitivity analysis revealed that the studies of Adly et al. (39) and Ozdemir et al. (47) had a greater influence on the results (Figure 7). After the exclusion of these two studies, the pooled sensitivity and specificity of sTREM-1 decreased from 0.94 to 0.93 and 0.87 to 0.81, respectively.

### Data analysis in prognostic value

**Figure 8:**
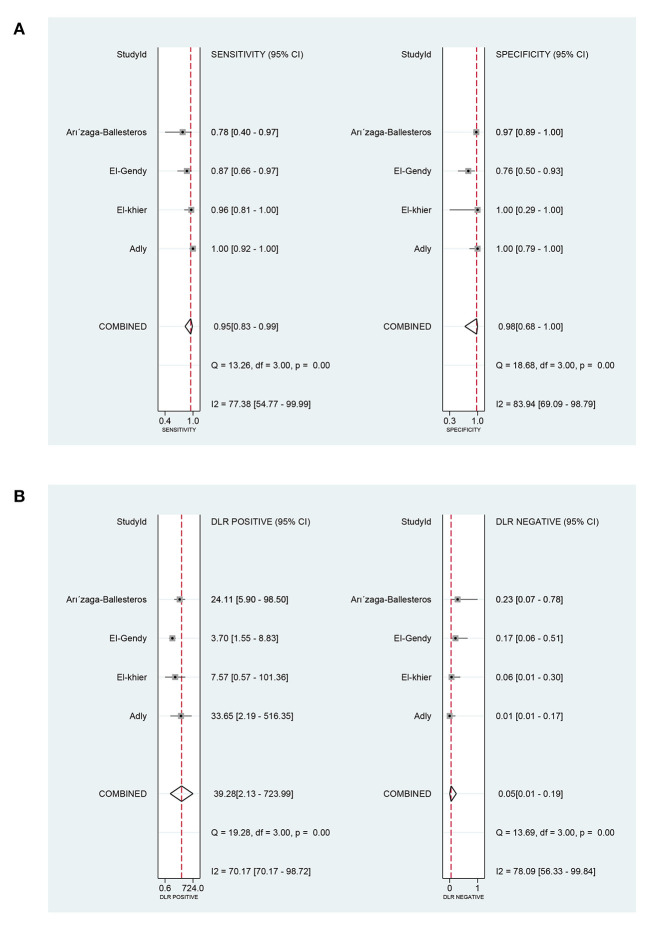
The pooled sensitivity, specificity, PLR, and NLR of the prognostic value of sTREM-1 in neonatal sepsis. **(A)** The pooled sensitivity and specificity. **(B)** The pooled PLR and NLR. PLR, positive likelihood ratio; NLR, negative likelihood ratio; sTREM-1, soluble triggering receptor expressed on myeloid cell-1.

In terms of the prognostic value of sTREM-1 in neonatal sepsis, the aggregate sensitivity and specificity were 0.95 (95% CI: 0.83, 0.99) and 0.98 (95% CI: 0.68, 1.00), respectively (Figure 8A). The pooled PLR and NLR were 39.28 (95% CI: 2.13, 723.99) and 0.05 (95% CI: 0.01, 0.19), respectively (Figure 8B). The DOR was 789.61 (95% CI: 17.53, 35560.72) and the area under the SROC curve was 0.99 (95% CI: 0.97, 0.99; Figure 4B).

The *I*^2^-test results for the pooled sensitivity and specificity were 77.38% (*P* < 0.05) and 83.94% (*P* < 0.05), respectively, indicating the existence of heterogeneity. Considering the small number of included studies, meta-regression analysis and subgroup analysis were not used to explore the heterogeneity caused by non-threshold effects.

The Deek's funnel plot of the included studies suggested there was no significant publication bias in the prognostic value of sTREM-1 (Figure 5B), *P* = 0.62). Sensitivity analysis showed that the results were stable and reliable (Supplementary Figure 1).

## Discussion

Neonatal sepsis is the third most common cause of neonatal death, which is also one of the major factors leading to neonatal disability (5). In that case, early diagnosis and treatment are essential to reduce mortality and disability rate. Currently, in view of the limitations of blood culture, clinical symptoms and signs, and laboratory indicators in the diagnosis of neonatal sepsis, there are no recommended biomarkers for the diagnosis of neonatal sepsis. The exploration of convenient and effective biomarkers for neonatal sepsis has become a research hotspot. The purpose of this study is to investigate the role of TREM-1 as a biomarker in the diagnosis and prognosis of neonatal sepsis.

The results of our meta-analysis including 10 studies (36, 38, 39, 41–47) manifested that the pooled sensitivity and specificity of sTREM-1 in the diagnosis of neonatal sepsis were 0.94 (95% CI: 0.82, 0.98) and 0.87 (95% CI: 0.70, 0.95), respectively. A previous meta-analysis by Bellos et al. showed that sTREM-1 as a predictor of neonatal sepsis had a pooled sensitivity of 0.95 (95% CI: 0.81, 0.99) and specificity of 0.87 (95% CI: 0.56, 0.97) (26), which further indicated that sTREM-1 had high sensitivity and specificity as a diagnostic biomarker. The area under the SROC curve summarized in our study was 0.96 (95% CI: 0.94, 0.98), indicating high accuracy of sTREM-1 in the diagnosis of neonatal sepsis. The heterogeneity of the non-threshold effect was mainly derived from the type of sample and study design, which was explored by meta-regression analysis and subgroup analysis. Subgroup analysis showed that the sensitivity of sTREM-1 in the diagnosis of neonatal sepsis was significantly different between the blood subgroup and the urine subgroup (*P* < 0.05), indicating that type of sample may affect the accuracy of the diagnostic test. In addition, the sensitivity of the urine subgroup was significantly lower than that of the blood subgroup (0.79 vs. 0.96), which may be due to the dilution of sTREM-1 during urine production (49). Meta-regression analysis indicated that the sensitivity of sTREM-1 was significantly different between the prospective and non-prospective studies (*P* < 0.05), suggesting that study design may affect the accuracy of diagnostic tests. However, this conclusion was not supported by strong evidence, because there was only one non-prospective study. More studies are needed to investigate the effect of study design on the accuracy of diagnostic tests.

In previous studies, Ghonai et al. found that TREM-1 mRNA had a moderate ability (AUC: 0.708) to diagnose neonatal sepsis, similar to its accuracy (AUC: 0.75) in the diagnosis of adult sepsis (48, 50). While, compared with the diagnosis of neonatal sepsis, TREM-1 mRNA had higher accuracy (AUC: 0.902 vs. 0.708) in predicting the mortality of neonatal sepsis (48). In addition, the expression level of TREM-1 mRNA in neonates with septic shock was significantly lower than that in EOS and LOS, and the difference was statistically significant (48). This indicated that TREM-1 mRNA expression level was negatively correlated with the severity of sepsis, which was consistent with the study from Atef et al. (48, 51). However, Tao et al. found that there were no differences in TREM-1 mRNA expression level between severe sepsis and septic shock groups (52). It should be noticed that the relationship between TREM-1 mRNA expression level and the severity of disease was still controversial, therefore, accurate conclusions could not be drawn. Besides, the mTREM-1 was also demonstrated to be a potential marker for the diagnosis of neonatal sepsis. In the study of Mazzucchelli et al., TREM-1 expressed on the surface of polymorphonuclear neutrophils was moderately associated with the diagnosis of neonatal sepsis (37).

The expression form of TREM-1 included in our meta-analysis was sTREM-1. It was inferred that the sTREM-1 form may be derived from the translation of the splicing variant of TREM-1 mRNA, or a proteolytic cleavage of the cell-surface anchored TREM-1 (16, 53, 54). Studies had found that two other forms of TREM-1, namely, the mRNA transcription level and the protein expression level of TREM-1 on the cell surface had a certain clinical value in the identification and/or prognosis of sepsis (50, 51, 55). In that case, we also qualitatively analyzed the role of TREM-1 mRNA and mTREM-1 in the diagnosis and/or prognosis of neonatal sepsis.

Our meta-analysis included four literature (39–41, 43) about the prognostic value of sTREM-1 in neonatal sepsis. It indicated that the pooled sensitivity, specificity and area under the SROC curve were 0.95, 0.98, and 0.99, respectively. These results suggested that sTREM-1 had high sensitivity and specificity in the prediction of prognosis of neonatal sepsis, which made it a creditable biomarker for distinguishing survival and death from neonatal sepsis.

From the evaluation results of the risk of bias, it was found that the high-risk items were mainly index test and patient selection. On the one hand, high risk of bias was found in the index test domain because in most studies the threshold value for TREM-1 expression to diagnose neonatal sepsis was determined by ROC curves rather than predetermined (36, 38, 39, 41–43, 45–48). On the other hand, the high risk of bias in patient selection was mainly because some of the studies were not continuous or random cases within a certain time range (36, 37, 39, 41). In the evaluation of applicability, two studies scored as high risk in the index test domain as they used TREM-1 mRNA or mTREM-1 as biomarkers, which limited their clinical applicability (37, 48). In general, the overall risk of bias was within the acceptable range and the applicability was high.

In recent years, the search for an ideal biomarker with sufficient diagnostic accuracy in neonatal sepsis is still ongoing. At present, biomarkers that have been studied extensively include procalcitonin (PCT), interleukin-6 (IL-6), presepsin, and serum amyloid A (SAA). In four meta-analysis studies, the pooled sensitivity of PCT, IL-6, presepsin, and SAA were 0.81, 0.76, 0.91, and 0.84, respectively, and the pooled specificity was 0.79, 0.79, 0.91, and 0.89, respectively (56–59). Obviously, the pooled sensitivity (0.95) of sTREM-1 was higher than that of PCT, IL-6, presepsin, and SAA, and the pooled specificity (0.87) was higher than that of PCT and IL-6. However, we cannot conclude that sTREM-1 has higher diagnostic efficacy than them in neonatal sepsis. Due to the influence of population selection, gold standard selection and sample selection time on sensitivity and specificity cannot be excluded. If multiple single biomarkers can be compared in the same study and the study design can be standardized, it may avoid the incorrect evaluation of the diagnostic performance of a single biomarker and increase the reliability of the results. No single biomarker was found to have sufficient diagnostic accuracy to diagnose neonatal sepsis (60). The combination of biomarkers may be a strategy to improve diagnostic accuracy (60). Therefore, we should systematically evaluate the independent diagnostic efficacy of sTREM-1, hoping to provide clinical evidence for sTREM-1 in combination with other sensitive biomarkers. Currently, there is no systematic review of the co-diagnosis of sTREM-1 in neonatal sepsis. Studies on the prognostic value and severity of sTREM-1 in neonatal sepsis are also relatively scarce. All of these provide a new direction for our future research.

This study has the following advantages: (1) this study is the first comprehensive study to investigate the role of TREM-1 expression in the diagnosis and prognosis of neonatal sepsis; (2) meta-regression analysis and subgroup analysis were used to explore the heterogeneity caused by non-threshold effect; (3) sensitivity analysis was used to evaluate the stability of the results of TREM-1 expression in the diagnostic and prognostic value of neonatal sepsis.

However, due to the limitations of meta-analysis, the deficiencies of this study are as follows: (1) the number of clinical studies on sTREM-1 as a biomarker of neonatal sepsis that could be included in the meta-analysis was relatively small; (2) the sample size of the included population in some studies was small, which may lead to selection bias (36, 37, 40–47); (3) the threshold value for the detection of TREM-1 expression to diagnose and evaluate the prognosis of neonatal sepsis was not set in advance, but the optimal threshold value was determined by ROC curve; (4) this study included all literatures published in English, so some research data may be omitted, affecting its comprehensiveness; (5) for the detection of TREM-1 expression in this study, differences caused by the technician's own technology, usage method, and surroundings could not be excluded; (6) included studies were mostly published by Egyptian scholars, which might have regional bias (39, 41, 43, 44, 46, 48); (7) in the review process, only English databases were retrieved, and no other language databases were retrieved. In addition, we manually retrieved three identified studies (41, 43, 44), which would lead to incomplete retrieval and certain selection bias.

## Conclusion

With a comprehensive analysis, sTREM-1 was demonstrated to be a credible biomarker for the diagnosis and prognosis of neonatal sepsis; TREM-1 mRNA and mTREM-1 may be potential markers for the diagnosis and/or prognosis of neonatal sepsis. Due to the limited number and quality of included studies, larger-scale and high-quality studies are still needed to improve diagnostic accuracy.

## Data Availability Statement

The original contributions presented in the study are included in the article/supplementary material, further inquiries can be directed to the corresponding authors.

## Author contributions

CC and HZ: conception and design of the research, analysis and interpretation of data, and drafting of the manuscript. QG and GD: performed the database search, acquisition of data, study selection, and data extraction. HZ and KL: statistical analysis and revision of the manuscript for important intellectual content. All authors approved the final manuscript as submitted and agree to be accountable for all aspects of the work.

## Funding

This work was supported financially by the funding of the Project of Science and Technology plan of the Jiangxi Health Committee (20204503), the Natural Science Foundation of Jiangxi Province (20202BAB216002), and the Key Research Project of Gannan Medical University (ZD201906).

## Conflict of interest

The authors declare that the research was conducted in the absence of any commercial or financial relationships that could be construed as a potential conflict of interest.

## Publisher's note

All claims expressed in this article are solely those of the authors and do not necessarily represent those of their affiliated organizations, or those of the publisher, the editors and the reviewers. Any product that may be evaluated in this article, or claim that may be made by its manufacturer, is not guaranteed or endorsed by the publisher.
