# Supplementary material for: Diagnostic and prognostic predictive values of triggering receptor expressed on myeloid cell-1 expression in neonatal sepsis: A meta-analysis and systematic review
Source: Front Pediatr. 2022 Jul 22;10:929665. doi: 10.3389/fped.2022.929665 (PMC9354627; doi:10.3389/fped.2022.929665)
Supplement: Supplementary file 1 [file Presentation_1.pdf]

Search Strategy

1. Embase

....

No.	Query Results	Results	Date
#8.	#2 AND #6 AND #7	208	12 Feb 2022
#7.	#3 OR #4	1,986	12 Feb 2022
#6.	#1 OR #5	277,141	12 Feb 2022
#5.	'neonatal sepsis':ab,ti OR 'newborn':ab,ti OR 'septic':ab,ti OR 'septicemia':ab,ti	273,629	12 Feb 2022
#4.	'soluble triggering expressed receptor on myeloid cells 1':ab,ti OR 'strem-1':ab,ti OR 'trem-1':ab,ti OR 'trem1 protein, human':ab,ti	1,232	12 Feb 2022
#3.	'triggering receptor expressed on myeloid cells 1'/exp	1,718	12 Feb 2022
#2.	[1966-2022]/py	38,634,143	12 Feb 2022
#1.	'newborn sepsis'/exp	9,221	12 Feb 2022

....

2. PubMed

Search number Query Sort By Filters Search Details Results Time

8 (((((newborn sepsis[Title/Abstract]) OR (newborn[Title/Abstract])) OR (septic[Title/Abstract])) OR (septicemia[Title/Abstract])) OR ("Neonatal Sepsis"[Mesh])) AND (("Triggering Receptor Expressed on Myeloid Cells-1"[Mesh]) OR ((((soluble triggering expressed receptor on myeloid cells 1[Title/Abstract]) OR (strem-1[Title/Abstract])) OR (trem-1[Title/Abstract])) OR (trem1 protein, human[Title/Abstract]))) from 1000/1/1 - 2022/2/12 (("newborn sepsis"[Title/Abstract] OR "newborn"[Title/Abstract] OR "septic"[Title/Abstract] OR "septicemia"[Title/Abstract] OR "Neonatal Sepsis"[MeSH Terms]) AND ("Triggering Receptor Expressed on Myeloid Cells-1"[MeSH Terms] OR (((("solubility"[MeSH Terms] OR "solubility"[All Fields] OR "solubilities"[All Fields] OR "soluble"[All Fields] OR "solubles"[All Fields] OR "solublization"[All Fields] OR "solublize"[All Fields] OR "solublized"[All Fields]) AND ("precipitating factors"[MeSH Terms] OR ("precipitating"[All Fields] AND "factors"[All Fields]) OR "precipitating factors"[All Fields] OR "trigger"[All Fields] OR "triggers"[All Fields] OR "triggerable"[All Fields] OR "triggered"[All Fields] OR "triggering"[All Fields] OR "triggerings"[All Fields]) AND ("express"[All Fields] OR "expresse"[All Fields] OR "expresses"[All Fields] OR "expressing"[All Fields] OR "expressions"[All Fields] OR "gene expression"[MeSH Terms] OR ("gene"[All Fields] AND "expression"[All Fields]) OR "gene expression"[All Fields] OR "expressed"[All Fields] OR "expression"[All Fields]) AND ("receptor"[All Fields] OR "receptor s"[All Fields] OR "receptors"[All Fields])) AND "on myeloid cells 1"[Title/Abstract]) OR "strem-1"[Title/Abstract] OR "trem-1"[Title/Abstract] OR ("trem1"[All Fields] AND "protein human"[Title/Abstract])))) AND (1000/1/1:2022/2/12[pdat]) 116 7:25:44

7 (((((newborn sepsis[Title/Abstract]) OR (newborn[Title/Abstract])) OR (septic[Title/Abstract])) OR (septicemia[Title/Abstract])) OR ("Neonatal Sepsis"[Mesh])) AND

(("Triggering Receptor Expressed on Myeloid Cells-1"[Mesh]) OR ((((soluble triggering expressed receptor on myeloid cells 1[Title/Abstract]) OR (strem-1[Title/Abstract])) OR (trem-1[Title/Abstract])) OR (trem1 protein, human[Title/Abstract]))) ("newborn sepsis"[Title/Abstract] OR "newborn"[Title/Abstract] OR "septic"[Title/Abstract] OR "septicemia"[Title/Abstract] OR "Neonatal Sepsis"[MeSH Terms]) AND ("Triggering Receptor Expressed on Myeloid Cells-1"[MeSH Terms] OR (((("solubility"[MeSH Terms] OR "solubility"[All Fields] OR "solubilities"[All Fields] OR "soluble"[All Fields] OR "solubles"[All Fields] OR "solublization"[All Fields] OR "solublize"[All Fields] OR "solublized"[All Fields]) AND ("precipitating factors"[MeSH Terms] OR ("precipitating"[All Fields] AND "factors"[All Fields]) OR "precipitating factors"[All Fields] OR "trigger"[All Fields] OR "triggers"[All Fields] OR "triggerable"[All Fields] OR "triggered"[All Fields] OR "triggering"[All Fields] OR "triggerings"[All Fields]) AND ("express"[All Fields] OR "expresse"[All Fields] OR "expresses"[All Fields] OR "expressing"[All Fields] OR "expressions"[All Fields] OR "gene expression"[MeSH Terms] OR ("gene"[All Fields] AND "expression"[All Fields]) OR "gene expression"[All Fields] OR "expressed"[All Fields] OR "expression"[All Fields]) AND ("receptor"[All Fields] OR "receptor s"[All Fields] OR "receptors"[All Fields])) AND "on myeloid cells 1"[Title/Abstract]) OR "strem-1"[Title/Abstract] OR "trem-1"[Title/Abstract] OR ("trem1"[All Fields] AND "protein human"[Title/Abstract]))) 116 7:10:38

6 ("Triggering Receptor Expressed on Myeloid Cells-1"[Mesh]) OR ((((soluble triggering expressed receptor on myeloid cells 1[Title/Abstract]) OR (strem-1[Title/Abstract])) OR (trem-1[Title/Abstract])) OR (trem1 protein, human[Title/Abstract])) "Triggering Receptor Expressed on Myeloid Cells-1"[MeSH Terms] OR (((("solubility"[MeSH Terms] OR "solubility"[All Fields] OR "solubilities"[All Fields] OR "soluble"[All Fields] OR "solubles"[All Fields] OR "solublization"[All Fields] OR "solublize"[All Fields] OR "solublized"[All Fields]) AND ("precipitating factors"[MeSH Terms] OR ("precipitating"[All Fields] AND "factors"[All Fields]) OR "precipitating factors"[All Fields] OR "trigger"[All Fields] OR "triggers"[All Fields] OR "triggerable"[All Fields] OR "triggered"[All Fields] OR "triggering"[All Fields] OR "triggerings"[All Fields]) AND ("express"[All Fields] OR "expresse"[All Fields] OR "expresses"[All Fields] OR "expressing"[All Fields] OR "expressions"[All Fields] OR "gene expression"[MeSH Terms] OR ("gene"[All Fields] AND "expression"[All Fields]) OR "gene expression"[All Fields] OR "expressed"[All Fields] OR "expression"[All Fields]) AND ("receptor"[All Fields] OR "receptor s"[All Fields] OR "receptors"[All Fields])) AND "on myeloid cells 1"[Title/Abstract]) OR "strem-1"[Title/Abstract] OR "trem-1"[Title/Abstract] OR ("trem1"[All Fields] AND "protein human"[Title/Abstract])) 980 7:10:22

5 (((soluble triggering expressed receptor on myeloid cells 1[Title/Abstract]) OR (strem-1[Title/Abstract])) OR (trem-1[Title/Abstract])) OR (trem1 protein, human[Title/Abstract])

((("solubility"[MeSH Terms] OR "solubility"[All Fields] OR "solubilities"[All Fields] OR "soluble"[All Fields] OR "solubles"[All Fields] OR "solublization"[All Fields] OR "solublize"[All Fields] OR "solublized"[All Fields]) AND ("precipitating factors"[MeSH Terms] OR ("precipitating"[All Fields] AND "factors"[All Fields]) OR "precipitating factors"[All Fields] OR "trigger"[All Fields] OR "triggers"[All Fields] OR "triggerable"[All Fields] OR "triggered"[All Fields] OR "triggering"[All Fields] OR "triggerings"[All Fields]) AND ("express"[All Fields] OR "expresse"[All Fields] OR "expresses"[All Fields] OR "expressing"[All Fields] OR "expressions"[All Fields] OR "gene expression"[MeSH Terms] OR ("gene"[All Fields] AND

"expression"[All Fields]) OR "gene expression"[All Fields] OR "expressed"[All Fields] OR "expression"[All Fields]) AND ("receptor"[All Fields] OR "receptor s"[All Fields] OR "receptors"[All Fields])) AND "on myeloid cells 1"[Title/Abstract]) OR "strem-1"[Title/Abstract] OR "trem-1"[Title/Abstract] OR ("trem1"[All Fields] AND "protein human"[Title/Abstract]) 848 7:10:08

4 "Triggering Receptor Expressed on Myeloid Cells-1"[Mesh] Most Recent "Triggering Receptor Expressed on Myeloid Cells-1"[MeSH Terms] 727 7:08:55

3 ((((newborn sepsis[Title/Abstract]) OR (newborn[Title/Abstract])) OR (septic[Title/Abstract])) OR (septicemia[Title/Abstract])) OR ("Neonatal Sepsis"[Mesh]) "newborn sepsis"[Title/Abstract] OR "newborn"[Title/Abstract] OR "septic"[Title/Abstract] OR "septicemia"[Title/Abstract] OR "Neonatal Sepsis"[MeSH Terms] 224,151 7:08:37

2 (((newborn sepsis[Title/Abstract]) OR (newborn[Title/Abstract])) OR (septic[Title/Abstract])) OR (septicemia[Title/Abstract]) "newborn sepsis"[Title/Abstract] OR "newborn"[Title/Abstract] OR "septic"[Title/Abstract] OR "septicemia"[Title/Abstract] 223,345 7:03:26

1 "Neonatal Sepsis"[Mesh] Most Recent "Neonatal Sepsis"[MeSH Terms] 1,075 7:01:35

3. Ovid

#1 (neonatal sepsis or newborn or septic or septicemia or newborn sepsis).ti,ab,kw.
#2 (triggering receptor expressed on myeloid cells 1 or soluble triggering expressed receptor on myeloid cells 1 or strem-1 or trem-1 or trem1 protein, human).ti,ab,kw.
#3 #1 and #2

4. Scopus

(TITLE-ABS-KEY ("neonatal sepsis" or "newborn" or "septic" or "septicemia" or "newborn sepsis") AND TITLE-ABS-KEY ("triggering receptor expressed on myeloid cells 1" or "soluble triggering expressed receptor on myeloid cells 1" or "strem-1" or "trem-1" or "trem1 protein, human"))

5. Web of science

#1 262,577 TS= (neonatal sepsis or newborn or septic or septicemia or newborn sepsis)
#2 1,518 TS= (triggering receptor expressed on myeloid cells 1 or soluble triggering expressed receptor on myeloid cells 1 or strem-1 or trem-1 or trem1 protein, human)
#3 #1 and #2

6. ProQuest

S1 AB, TI ("neonatal sepsis" or "newborn" or "septic" or "septicemia" or "newborn sepsis") 90594
S2 AB, TI ("triggering receptor expressed on myeloid cells" or "soluble triggering expressed receptor on myeloid cells 1" or "strem-1" or "trem-1" or "trem1 protein, human") 305
S3 AB, TI ("neonatal sepsis" or "newborn" or "septic" or "septicemia" or "newborn sepsis") AND AB, TI ("triggering receptor expressed on myeloid cells" or "soluble triggering expressed receptor on myeloid cells 1" or "strem-1" or "trem-1" or "trem1 protein, human") 33

7. Cochrane Library

With Cochrane Library publication date to Feb 2022.

#1 neonatal sepsis Mesh 2512

#2 (newborn):ab,ti,kw OR (septic):ab,ti,kw OR (septicemia):ab,ti,kw OR (newborn sepsis):ab,ti,kw 33937

#3 #1 or #2 34858

#4 triggering receptor expressed on myeloid cells 33

#5 (soluble triggering expressed receptor on myeloid cells 1):ab,ti,kw OR (strem-1):ab,ti,kw OR (trem-1):ab,ti,kw OR (trem1 protein, human):ab,ti,kw 51

#6 #4 or #5 56

#7 #3 and #6 17

Literatures from other sources

Three literatures were retrieved manually.

1. Serum Triggering Receptor Expressed on Myloid Cells-1 (sTREM-1) and its Role in Diagnosis of Neonatal Sepsis.
2. Value of Measurement of sTREM-1 Factor in Diagnosis and Prognosis of Neonatal Sepsis.
3. Soluble triggering receptor expressed on myeloid cells-1 (sTREM-1) as a diagnostic and prognostic marker of late-onset sepsis in preterm neonates.
