# Supplementary material for: Diagnostic and prognostic predictive values of triggering receptor expressed on myeloid cell-1 expression in neonatal sepsis: A meta-analysis and systematic review
Source: Front Pediatr. 2022 Jul 22;10:929665. doi: 10.3389/fped.2022.929665 (PMC9354627; doi:10.3389/fped.2022.929665)
Supplement: Supplementary file 2 [file Table_1.docx]

**Figure legends**

**Supplementary Table 1.** Results of subgroup analysis. Abbreviations: No, number.

**Supplementary Figure 1.** Sensitivity analysis of in the prognostic value of TREM-1 expression in neonatal sepsis. Abbreviations: TREM-1, triggering receptor expressed on myeloid cell-1.

**Supplementary Table 1.** Results of subgroup analysis.

| Subgroup | | No. | Sensitivity | Specificity |
| --- | --- | --- | --- | --- |
| Type of sample | blood sample | 8 | 0.96  （95%CI:0.91,1.00） | 0.88  （95%CI:0.75,1.00） |
|  | not blood sample | 2 | 0.79（95%CI:0.41,1.00） | 0.82（95%CI:0.53,1.00） |
| Study design | prospective study | 9 | 0.92（95%CI:0.84,1.00） | 0.83（95%CI:0.71,0.96） |
|  | not prospective study | 1 | 1.00（95%CI:1.00,1.00） | 1.00（95%CI:1.00,1.00） |
| Sample size | ≥60 | 6 | 0.96（95%CI:0.89,1.00） | 0.91（95%CI:0.79,1.00） |
|  | ＜60 | 4 | 0.92（95%CI:0.77,1.00） | 0.79（95%CI:0.53,1.00） |
