# Supplementary material for: Diagnostic and prognostic predictive values of triggering receptor expressed on myeloid cell-1 expression in neonatal sepsis: A meta-analysis and systematic review
Source: Front Pediatr. 2022 Jul 22;10:929665. doi: 10.3389/fped.2022.929665 (PMC9354627; doi:10.3389/fped.2022.929665)
Supplement: Supplementary file 4 [file Table_3.docx]

Three literatures were retrieved manually.

1. Serum Triggering Receptor Expressed on Myloid Cells-1 (sTREM-1) and its Role in Diagnosis of Neonatal Sepsis.
2. Value of Measurement of sTREM-1 Factor in Diagnosis and Prognosis of Neonatal Sepsis.
3. Soluble triggering receptor expressed on myeloid cells-1 (sTREM-1) as a diagnostic and prognostic marker of late-onset sepsis in preterm neonates.
