# Supplementary figures and images for: Diagnostic and prognostic predictive values of triggering receptor expressed on myeloid cell-1 expression in neonatal sepsis: A meta-analysis and systematic review
Source: Front Pediatr. 2022 Jul 22;10:929665. doi: 10.3389/fped.2022.929665 (PMC9354627; doi:10.3389/fped.2022.929665)

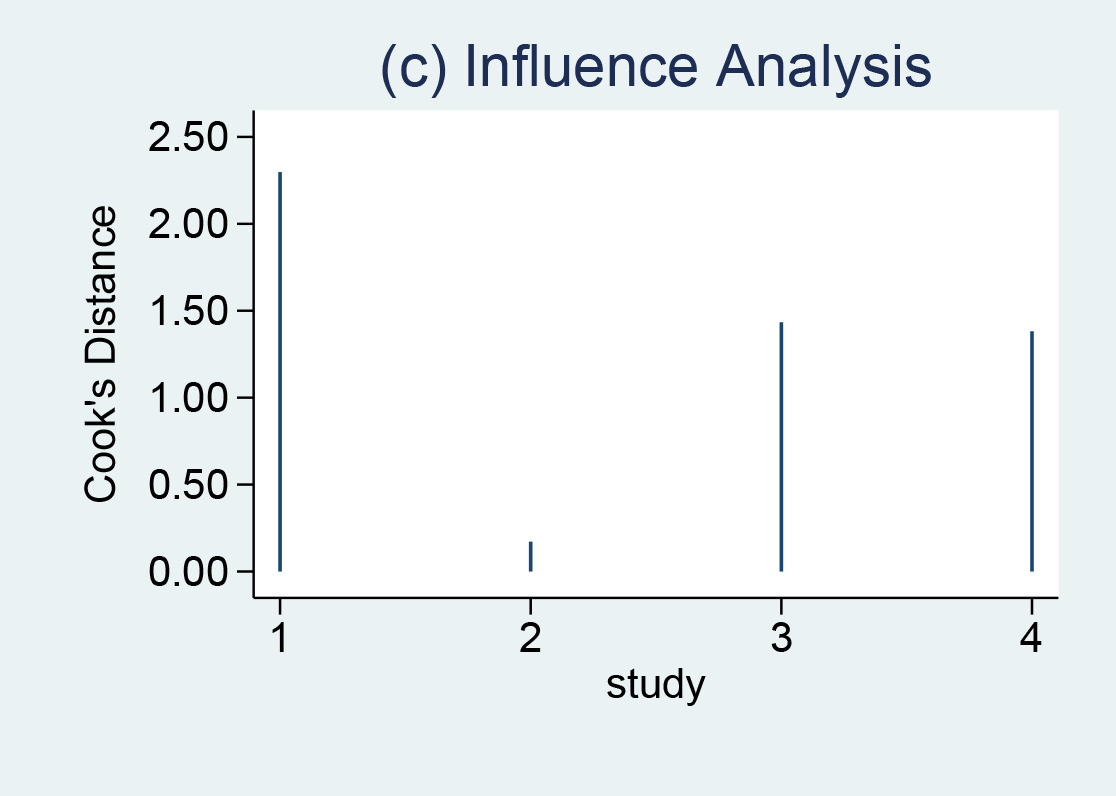

Supplement: Supplementary file 5 [file Image_1.TIF]

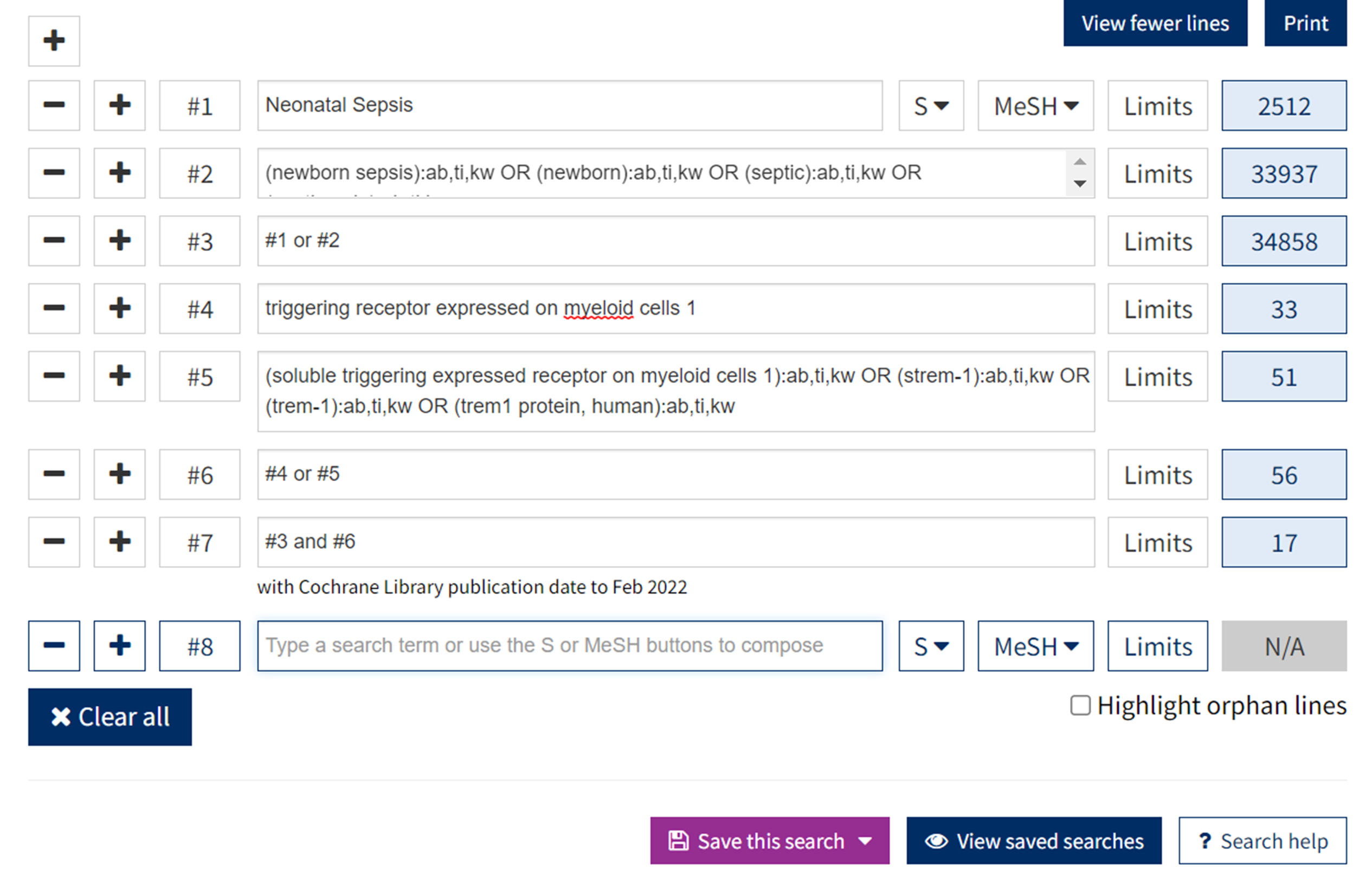

Supplement: Supplementary file 6 [file Image_2.PNG]

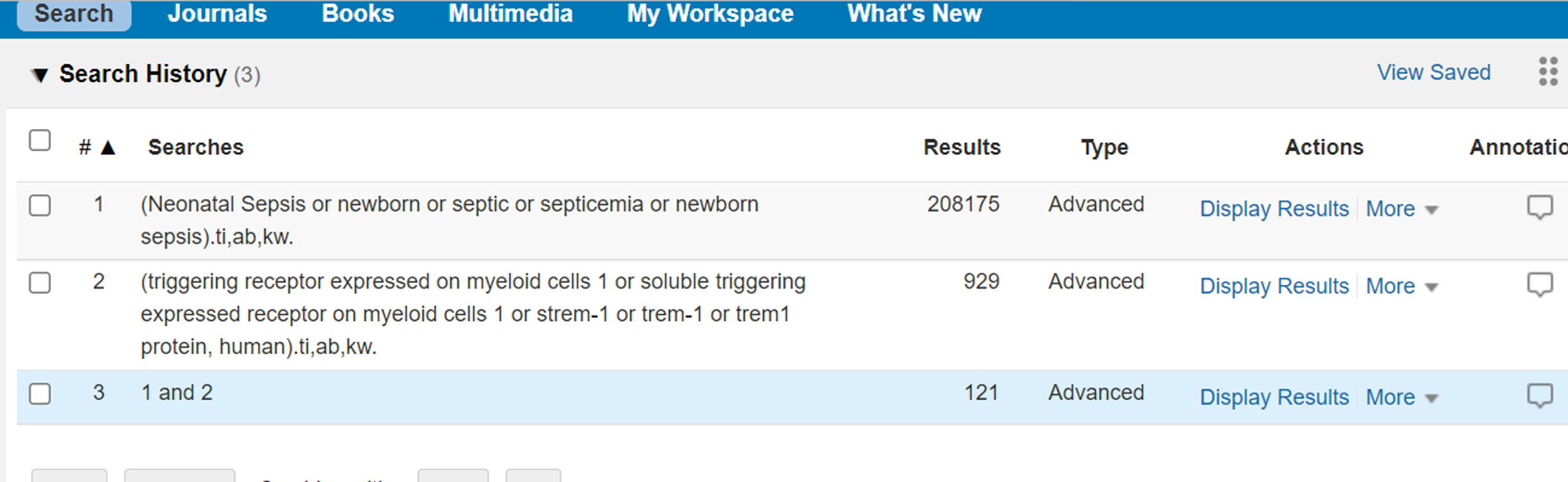

Supplement: Supplementary file 7 [file Image_3.PNG]

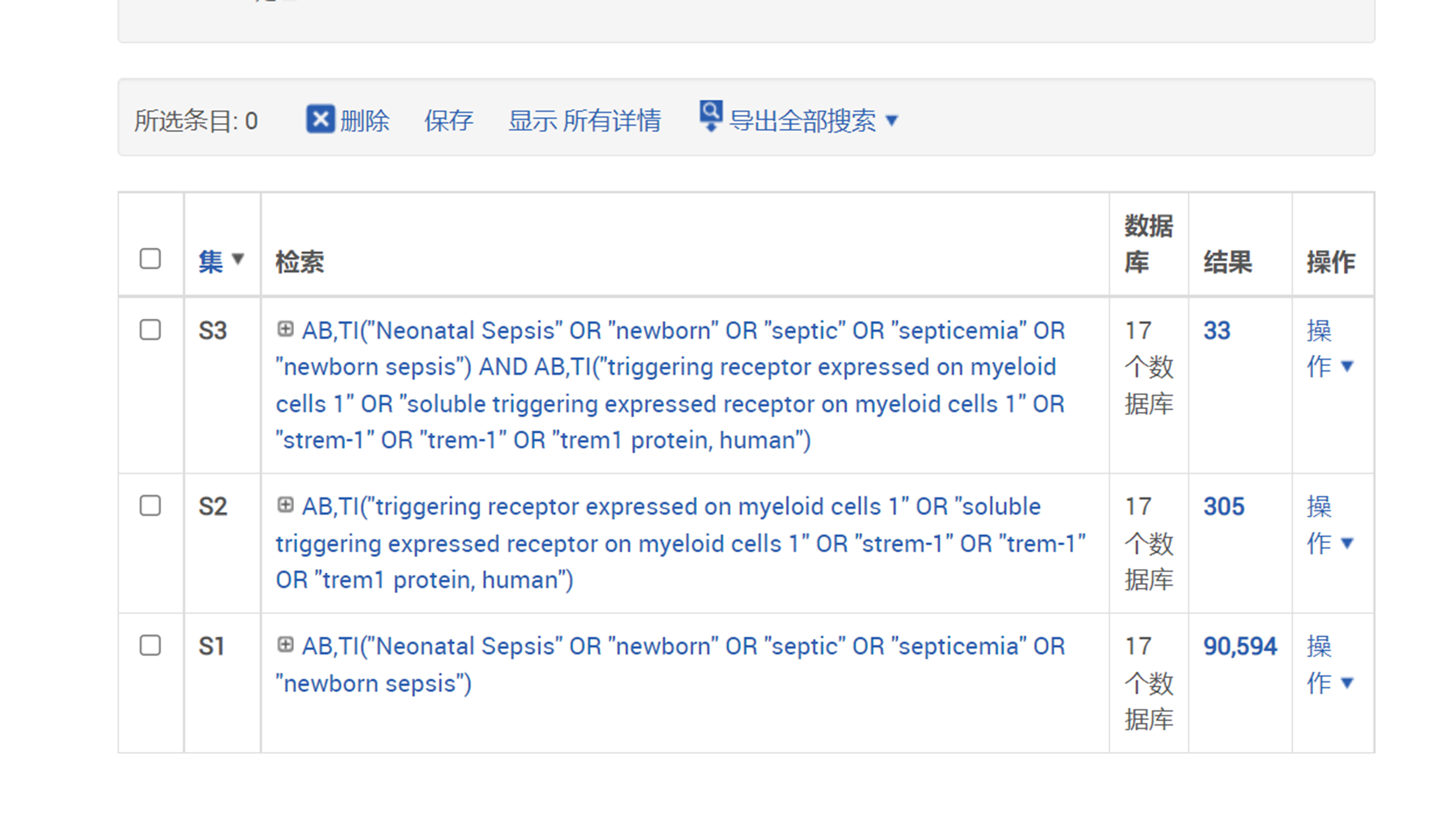

Supplement: Supplementary file 8 [file Image_4.PNG]

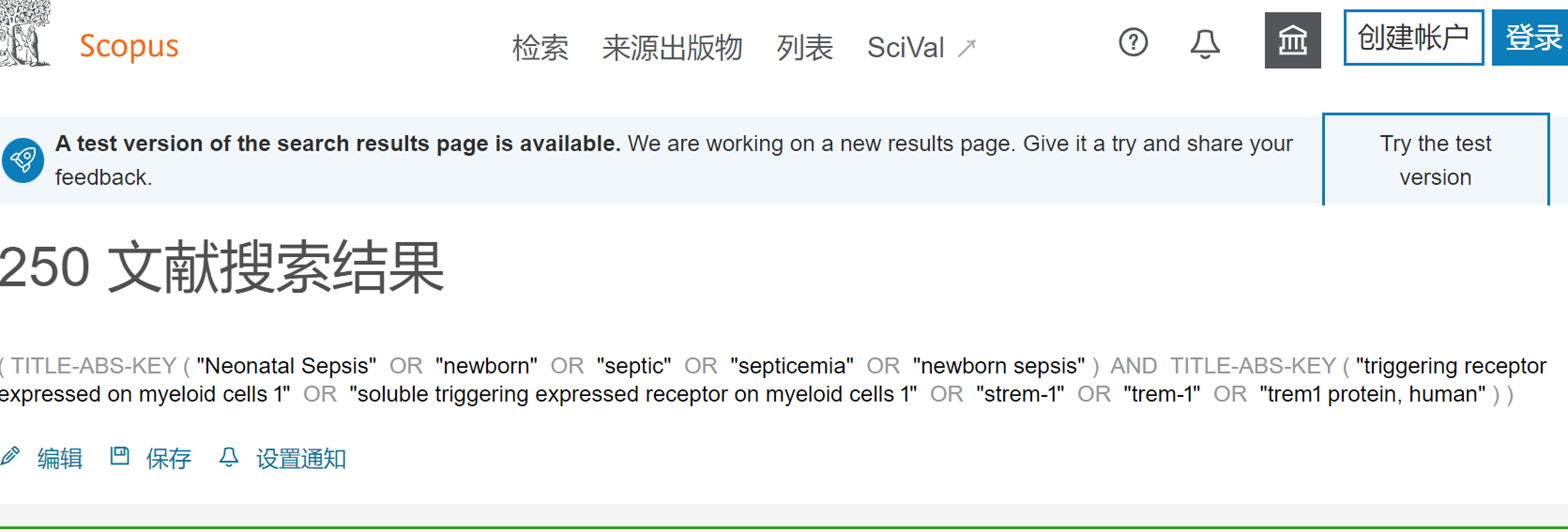

Supplement: Supplementary file 9 [file Image_5.PNG]

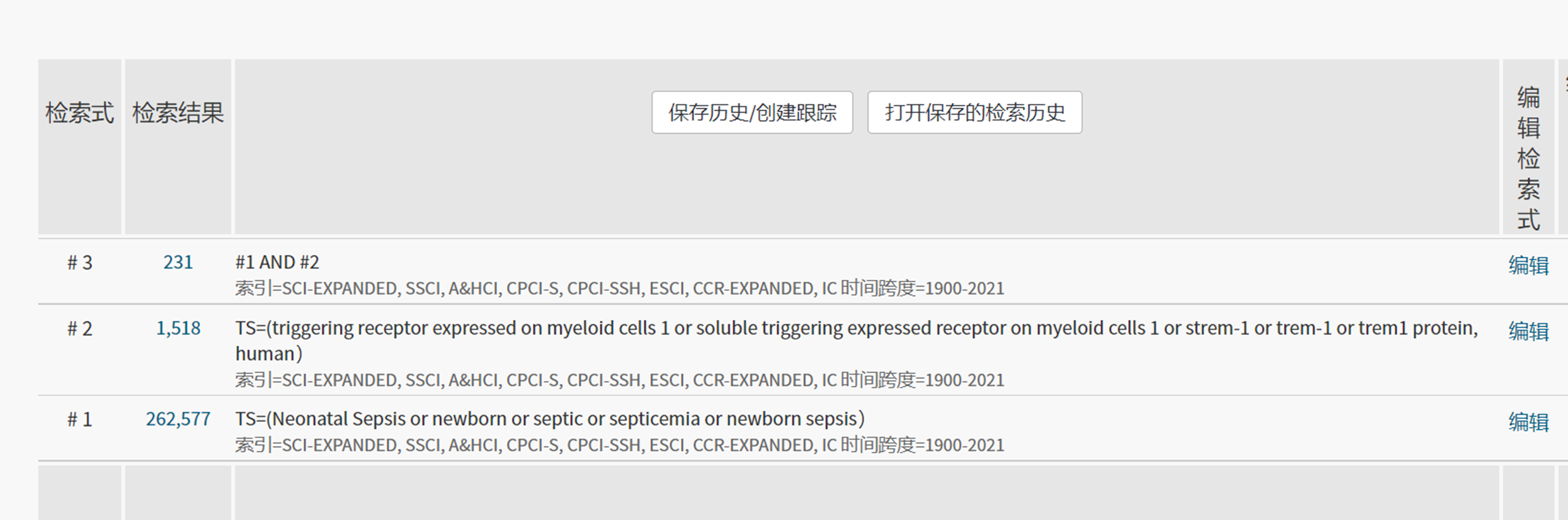

Supplement: Supplementary file 10 [file Image_6.PNG]
